# The Response to and Impact of the Ebola Epidemic: Towards an Agenda for Interdisciplinary Research

**DOI:** 10.15171/ijhpm.2017.104

**Published:** 2017-09-03

**Authors:** Michael Calnan, Erica W. Gadsby, Mandy Kader Kondé, Abdourahime Diallo, Jeremy S. Rossman

**Affiliations:** ^1^SSPSSR, University of Kent, Kent, UK.; ^2^Centre for Health Services Studies, University of Kent, Kent, UK.; ^3^Département Santé Publique, Université UGAN Conakry and FOSAD Health and Sustainable Development Foundation and CEFORPAG Center of Excellence for Training, Research on Malaria & Priority Diseases in Guinea, Conakry, Guinea.; ^4^FOSAD Health and Sustainable Development Foundation and CEFORPAG Center of Excellence for Training, Research on Malaria & Priority Diseases in Guinea, Conakry, Guinea.; ^5^School of Biosciences, University of Kent, Kent, UK.

**Keywords:** Ebola, Guinea, Research Priorities, Survivors, Social Impact

## Abstract

**Background:** The 2013-2016 Ebola virus disease (EVD) epidemic in West Africa was the largest in history and resulted in a huge public health burden and significant social and economic impact in those countries most affected. Its size, duration and geographical spread presents important opportunities for research than might help national and global health and social care systems to better prepare for and respond to future outbreaks. This paper examines research needs and research priorities from the perspective of those who directly experienced the EVD epidemic in Guinea.

**Methods:** The paper reports the findings from a research scoping exercise conducted in Guinea in 2017. This exercise explored the need for health and social care research, and identified research gaps, from the perspectives of different groups. Interviews were carried out with key stakeholders such as representatives of the Ministry of Health, non-governmental organizations (NGOs), academic and health service researchers and members of research ethics committees (N=15); health practitioners (N=12) and community representatives (N=11). Discussion groups were conducted with male and female EVD survivors (N=24) from two distinct communities.

**Results:** This research scoping exercise identified seven key questions for further research. An important research priority that emerged during this study was the need to carry out a comprehensive analysis of the wider social, economic and political impact of the epidemic on the country, communities and survivors. The social and cultural dynamics of the epidemic and the local, national and international response to it need to be better understood. Many survivors and their relatives continue to experience stigma and social isolation and have a number of complex unmet needs. It is important to understand what sort of support they need, and how that might best be provided. A better understanding of the virus and the long-term health and social implications for survivors and non-infected survivors is also needed.

**Conclusion:** This study identified a need and priority for interdisciplinary research focusing on the long-term sociocultural, economic and health impact of the EVD epidemic. Experiences of survivors and other non-infected members of the community still need to be explored but in this broader context.

## Background

In 2013, Guinea was the first country in West Africa to experience the recent outbreak of the Ebola virus disease (EVD) which as a whole resulted in over 28 000 cases and 11 000 deaths in 10 countries, making it the largest Ebola outbreak ever recorded.^1^ The epidemic took considerable time to contain, despite the extensive mobilisation of personnel, equipment and resources by national and international agencies.^2,3^

Viral, health and epidemiological factors alone do not appear to account for this difficulty in controlling the outbreak.^4^ It has been suggested that some of the social conditions that contributed to the size, extent and spread of the epidemic in Guinea and surrounding countries included war, population growth, poverty and a poor health infrastructure.^5^ These social conditions might be reflected in the relatively low life expectancy rates in Guinea, which stood at 59 years in 2015.^6^ Certainly, the capacity of the health system in Guinea appeared to be weak at the time of the outbreak, with several essential functions not performing well.^7,8^ It was reported that there were inadequate numbers of qualified health workers; infrastructure, logistics, health information, surveillance, governance and drug supply systems were weak; the organisation and management of health services was sub-optimal; and government health expenditure was low whereas private expenditure (mostly in the form of direct out-of-pocket payments for health services) was relatively high.^9^

In addition to health system weaknesses, one of the major barriers to controlling the disease appeared to be community resistance to the Ebola response.^10^ For example, the World Health Organization (WHO) reported, in a 6-month retrospective analysis on the first cases of the outbreak, they were sometimes met with violence from a fearful population.^11^ The communities’ fear appeared to be in response to the way intervention programs had been introduced.^12^ It also appeared to be due, in part, to the nature of the disease itself, which, as with other infectious diseases, disrupted the traditional cultural customs and behavioural practices for caring for the sick and dealing with a dying – or the death of – a relative, friend or member of the community.^5^

The scale of the emergency in West Africa was such that the international response has progressed through three phases^1^: (1) The rapid scale-up of the response, which included increasing the number of Ebola treatment centres, hiring and training teams in safe and dignified burials, and strengthening social mobilisation capacities. During this time, a United Nations (UN) Mission for Ebola Emergency Response was launched. (2) The strengthening of capacities for case finding and contact tracing, and the engagement of communities. During this time, clinical trials of Ebola vaccines and anti-viral therapeutics were initiated in Guinea which demanded considerable capacity with regards to ethics and governance, communication, surveillance, laboratory testing and trial management. And (3) the interruption of all remaining chains of Ebola transmission, which entailed: enhancing the rapid identification of all cases, deaths and contacts; establishing and maintaining safe triage and health facilities; building multi-disciplinary rapid response teams at regional and zone levels; providing incentives for individuals and communities to comply with public health measures; engaging in community-owned local response activities; improving Ebola survivor engagement and support; and ending human-to-human transmission of EVD in the populations and communities of the affected countries. This emergency response – particularly the last phase – was complemented by the joint West African government-led Ebola Recovery Assessment programme which aimed to lay the foundation for short, medium and long-term recovery. The focus in this programme was on four areas: health, nutrition and water, sanitation and hygiene; governance, peace building and social cohesion; infrastructure and basic services and socio-economic revitalisation.^13^

It has been recognised that outbreaks of emerging infectious diseases are sources of instability, uncertainty and sometime crises.^14,15^ There has been some sociological and political analysis of the way the Ebola epidemic was constructed as a problem or crisis outside Africa in high income countries,^3^ and how it became a global political as well as a health event.^2^ This analysis has tended to emphasise the importance of the influence of the international agencies in shaping the response but also the moral discourse or panic associated with this response.^3^ The role played by the global media has been highlighted, for instance, in enhancing the stigmatisation of those directly or indirectly linked with the outbreak.^3^ However, much of this research has been carried out ‘at a distance’ and there is limited detailed research evidence about the local and national responses to the EVD epidemic, and consequent missed opportunities to improve policy and practice responses in the future.^10,16^ There is also increasing recognition of the need for interdisciplinary research to examine the social dimensions of the epidemic, the policy response to it, the communities’ reactions to the response and how these factors intersected with the biological transmission of the virus, physical containment measures and community medical treatment.^2,17^

This paper addresses the lack of detailed analytical research to date on the perceptions and needs of those with direct experience of the Ebola epidemic in Guinea. It presents evidence from a study exploring research needs from the perspective of a number of key groups, including members of local communities. The original aim of the exercise was to identify priorities for health and social care research with and for survivors of EVD in Guinea. Survivors’ experiences have been the subject of limited previous research in Guinea which highlighted the stigma associated with Ebola and the consequences of social isolation for the mental health of survivors.^18,19^ The aim was to see if this was still a priority from the perspective of survivors and/or if there are other research questions that might need to be explored particularly in relation to the long term experiences of survivors and their family and communities. The objectives were: to explore survivors’ experiences of their various interactions with health, care and associated services delivered by local, national and international providers and agencies, including non-governmental organizations (NGOs); and to explore and discuss the need for health and social care research, and identify research gaps and priorities, from the perspectives of different groups – men, women, EVD survivors, community leaders, health practitioners, traditional healers, and local and national government stakeholders.

## Methods

The study followed a structured, participatory, inclusive approach guided by the principles and values of the Essential National Health Research (ENHR) strategy for priority setting.^20^ These principles include placing country priorities first; working towards equity in health; and linking research to action for development. The ENHR strategy, developed by the Commission on Health Research for Development, advocated the use of a systematized approach to priority setting that involved all stakeholders. The Council on Health Research for Development (COHRED) – established to assist with the implementation of this strategy – recommended a three stage approach (planning the priority-setting process, setting the priorities, and implementing the priorities) to increase the effectiveness of the priority-setting process.^21^ Since then, several WHO committees,^22,23^ and the Global Forum for Health Research,^24,25^ amongst others, have further elaborated methods, tools and frameworks for research priority setting, that are underpinned by the principles and values of the ENHR.

The study reported in this paper was a preliminary rapid assessment, rather than a full research prioritisation exercise. Due to time and resource constraints, it required a pragmatic approach guided by established conceptual frameworks for compiling information relevant for investigating health research priorities. The Combined Approach Matrix in particular guided us to explore not just the public health dimension (in terms of the magnitude of the problem, determinants and present level of knowledge), but also the institutional dimension (including the individual, household and community, health sector and sectors other than health), and the equity dimension (in particular gender, poverty and survivor status).^25^ The starting point for this work was that, in the specific area of EVD research, whilst investment was (at least initially and understandably) prioritised towards biomedical scientific research aimed at treating and preventing infection, it is likely that there are a number of areas where research and development could make an important difference to global health, but which are currently not recognised or not receiving appropriate attention (and resources).

The preparatory work for this study included the identification of key stakeholders, the collation and analysis of background information, and discussions with a range of interdisciplinary experts in health systems and policy research in Guinea, and in EVD research. The field visit included public engagement activities that enabled us to progress four elements of the ENHR process: getting to know the stakeholders; situation analysis/stocktaking; identification of research priority areas; and discussion and ranking of identified research priority areas. The goals of the public engagement activities were to become better informed about a range of people’s views and concerns about EVD research, to hear different perspectives and insights, and to become more sensitive to the social and ethical issues that relate to it. The aim was also to develop collaboration with stakeholders in Guinea, where research questions could be developed and explored in partnership with the public.

### Data Collection and Sampling

Data collection consisted of face-to-face interviews and focus group discussions. The purposive sample of key stakeholders (N = 15) selected for interviews included representatives from the Ministry of Health (N = 5), NGOs (N = 4), academic and health service researchers (N = 4) and members of ethics and research committees (N = 2). These data were complemented by interviews with health practitioners (N = 12, of which 2 were traditional healers) and community representatives (N = 11) and focus group discussions with male (N = 12) and female (N = 12) EVD survivors from two distinct communities in Guinea. Both communities were small townships. Site one was approximately 50 km from the capital (Conakry), and was affected towards the end of the epidemic. Site two was in the more remote, forest region of Guinea, and was within the prefecture where the first cases were identified in 2013.

Questions posed in interviews and discussions varied according to participants, and on information gathered during the field visit. However, they included questions to elicit information on: health status and social position (eg, information on the main health and health-related/social needs of people who have survived EVD, how these needs have changed over time, and the extent to which these needs are understood by others); health and social care systems (eg, the services available for local people, particularly in relation to needs expressed); health and social care research programmes (eg, awareness of and involvement in research for or with EVD survivors); and needs and values of survivors and other key stakeholders (eg, most important issues related to life after the EVD outbreak, now and in the future).

### Fieldwork and Analysis

The analysis made pragmatic compromises between timeliness and resource requirements and scientific rigour and validity. It drew on the technique of rapid appraisal, seeking to gain community perspectives of local health and social needs and to translate these findings into action.^26-29^ Data collected from one source were validated or rejected by checking with data from at least two other sources or methods of data collection. The majority of the interviews and discussion groups were recorded and notes were taken on the content and conduct of discussions. The interviews with key stakeholders were mainly carried out in French, and translated to English during the course of the discussion. The discussion with survivors at the two sites were conducted in two groups – one male, one female – and facilitated by a French speaker and a helper from the local community who spoke the local language. All were experienced facilitators and all participants contributed to the discussion. They used the same discussion guide for both groups. Both groups lasted for approximately one and a half hours.

The analysis was conducted iteratively within the research group (which constitutes the authors of this paper), through reviewing and summarising audio files and field notes, by identifying and sorting key themes, and by comparing and contrasting different perspectives. The researchers took particular note of, and sought to explore further, issues associated with equity in health. The analysis was limited by the multiple languages used within the data. A more complete analysis, involving the full translation and thematic coding of transcripts in a single language, would likely uncover further depth and nuance within the data. This paper is based on an initial descriptive analysis of salient themes which emerged from the interviews and discussions based on the field notes and summaries. It does not, therefore, contain direct quotes. The field work as a whole was carried out in Guinea in January 2017.

### Key Stakeholders’ Ranking of Research Priorities

The final phase of the research scoping exercise involved a presentation and discussion of findings to a meeting of the key stakeholders in Conakry, and (separately) to a meeting of key stakeholders in the more remote site two. The group of key stakeholders in Conakry did not include representatives from survivors’ groups, but did include participants with an in-depth understanding of the issues faced by survivors in Guinea. In site two, the group of key stakeholders included leaders of survivors’ groups. The research team proposed and explained the key themes that arose from the scoping exercise, emphasising the links between research and action for development. The stakeholders discussed these themes within the group setting, and were then asked independently to rank the topics in order of priority, according to their own perspectives and interests. No attempt was made at this stage to develop consensus within the group, and no temporal or financial parameters were defined. This allowed the research team to see how priorities of different stakeholders varied, and to rank the questions in order of averaged priority. It is important to acknowledge that priorities will change over time, and that research priorities can sometimes be individual. In this exercise, explicit criteria for the ranking exercise were not set.

## Results

This section describes the themes that arose in the interviews and discussion groups. They provide the basis for the research agenda set out in the final discussion section.

###  The Ebola Virus Disease Survivors

The initial focus of this research scoping exercise was on the survivors’ experiences. In group discussions, the survivors described the ways in which they and their lives had been affected by EVD. The issues that arose, clustered into key themes, are summarised in table one. The discussions showed that the social and economic implications of experiencing the virus were as important as the implications for physical health. Some of the concerns already noted in the literature about survivors’ experience were reinforced. For example, being stigmatised and excluded from the family and the community, and feeling lonely and isolated due to family break up were common sentiments expressed by both men and women. There were also major economic implications such as losing jobs and accommodation and generally suffering a serious reduction in income. Experiencing this illness and its consequences, perhaps not surprisingly, also had serious implications for well-being, happiness and mental health. There was a clear indication that these psychological needs were not being met (Table).

**Table:** Summary of Key Issues Raised by Men and Women in Group Discussions (in Both Sites)

**Key Themes**	**Men**	**Women**
Family	• Isolation from family activities • Families broken up/divided • Loss of spouse and/or other relatives • Stigma (rejected by or treated differently by the family)	• Stigma (being rejected by family) • Family breakup/abandonment by parent • Concern about meeting the needs of their children • Loss of many members of the family • Changed behaviours towards survivors (eg, separate utensils, keeping distance)
Relationships	• Stigmatisation • Difficulties with re-integrating into community • Not allowed access to toilets • Difficulty in finding a partner • Feeling alone • Keeping secrets (because of the fear of stigma) • Using isolation as a way of coping	• Victim blaming • Stigma (being excluded / rejected, being rejected by friends, isolation, in school) • Enforced migration • Suspicion amongst family members that any future illness may be Ebola again
Health	Frequent health problems including: • Joint pain/problems • Trembling • Fever • Insomnia • Memory problems • Vision problems • Fatigue	Varied health problems including: • Stomach pains • Joint pain/problems • Head aches • Frequent colds • Fatigue
Economics	• Loss of rented accommodation • Loss of work • Affected professional life • Loss of property (land, house) • Restriction of economic activities • Reduction of income	• Reduced income (sometimes because of loss of clients) • Loss of or reduced income generating activities • Loss of money • Loss of accommodation • Increase in debt • Loss of family property • Reduced performance at work (related to health issues)
Wellbeing	• Traumatised • Lack of consideration of the psycho-social needs of survivors • Difficulties in forgetting the past • Isolating oneself • Stress • Unhappiness	• Considering oneself a half-person • Unhappiness • Feeling pitied

There was a great deal of consistency in the issues raised by men and women, and within the two very different communities. However, in site two, more stories were heard about very large numbers of people dying within single families and villages than in site one. This is likely because the outbreak went undetected here for a while before infection control and containment measures were put in place. Both the men and women in site two identified the circulation of false rumours as a particular issue.

Survivors in all groups were worried about their health and what the virus is still doing to them. There was no clear understanding of virus persistence in their body, and anxiety and confusion about the lingering effects of the initial infection. Survivors reported still experiencing a number of symptoms – many bordering between mental and physical health. For example, whilst many reported experiencing fatigue, it is not known whether this was as a result of ongoing effects of the virus, or as a result of depression or post-traumatic stress. The support provided for physical health symptoms varied. Participants in certain research studies (eg, the ‘Postebogui’ study^18^) were able to get free healthcare. However, many of the survivors in the discussion groups expressed the need for help with their medical charges. In addition, informants told us that there was no formal support provided for mental healthcare, even for those involved in the research studies. This appears to be very important as many survivors in the discussion groups faced considerable difficulty obtaining informal support from the community as a result of the stigmatisation that they faced. Isolation and mental ill-health were sometimes extreme – leading to two recent suicides amongst the survivors in site two. Survivors’ groups organised by the local authorities were able to provide informal peer support to a limited degree, but this was hindered by difficulties with geographical spread and communication, and a lack of financial support to maintain the network.

The wider social and financial needs that survivors faced were met to a certain extent following discharge from the treatment centre. They received food donations from the World Food Programme, financial assistance from National Coordination Ebola response, international agencies and local authorities and NGOs, and free healthcare and other material support for short periods. However, some survivors expressed concern that not all the donations were reaching the survivors and the communities as intended. The support, whilst appreciated and beneficial, was short-lived compared with the ongoing needs of the survivors. Participants emphasised their desire to live and work independently rather than rely on other, external, hand-outs. They talked about their need for education and capacity building, both to become more literate, and to open opportunities for employment.

Most of the survivors in the discussion groups were aware of, or active participants in, some form of research on EVD. Many of them had signed up to give blood and/or semen samples for scientific analysis. Some had conducted health questionnaires. However, participants explained that they had not been involved in any research that asked them about their experiences either being infected or of the treatment they received, or the impact, for them, of having survived the virus.

### Health Practitioners and Community Representatives

There were many common themes expressed by both health practitioners and community representatives in both site one and site two. All the informants talked about the experiences and needs of survivors in much the same way as the survivors themselves – indeed many of the informants were themselves survivors. In addition, the informants described the ways in which their communities had been affected, and the ways in which their communities responded, both to the outbreak itself, and to the authorities’ response to the outbreak. It was clear that community representatives saw whole communities as having been profoundly affected by the outbreak, and the notion of communities surviving the experience, in addition to individuals surviving the virus, began to emerge. The key themes are outlined below, in no order of priority:

### Misunderstandings/Trust

There were many rumours surrounding Ebola, particularly with regards to where it came from and how it was spread. Negative reactions from some communities to the authorities’ (including government, local/international agencies and NGOs) response were triggered by a lack of understanding which seemed to emerge both from the initial message from authorities that Ebola cannot be cured, and from the practices of those engaged in the response (eg, spraying of areas, secure burials). For example, the communities’ perception of the ineffectiveness of treatment was reinforced by the fact that health practitioners – including both traditional healers and those practising scientific medicine – contracted the disease, and sometimes died from it. This was particularly the case in site two, since communities in this region were affected much earlier than elsewhere in the country. False rumours have been pervasive, damaging and lasting. These misunderstandings have contributed to a lack of trust not just with the authorities, but also with medical practitioners. Because of this, traditional healers played an important role (particularly in the early stages of the outbreak), where they received people who did not have trust/confidence in professional healers. The lack of trust that emerged as a result of misunderstandings extended to neighbours and communities and has persisted, resulting in the survivors facing stigma and discrimination. Sometimes, the misunderstanding and fear were such that individuals and groups acted in extreme ways towards each other, for example, by burning the house and possessions of survivors. Several health practitioners who themselves survived the virus reported difficulties in re-integrating at work, due to a lack of trust from their colleagues and patients.

### Fractured Communities

The impact of the epidemic and the response to it appeared to have fractured communities. Whilst this was due in part to misunderstandings, it partly arose as a consequence of the need to break cultural traditions and social norms (such as caring for relatives who are sick or visiting friends when sick) in order to break the chain of transmission. This enforced separation and created discord within families, villages and larger community groups. Sometimes, family separation was caused by the social and practical necessities of caring for children who had lost one or both parents to the disease. This itself was complicated by extreme economic hardship, which sometimes forced difficult decisions to be made – for example, where a surviving parent lost their income and felt no longer able to look after their child(ren). The social cohesion that was affected during the outbreak appears to be taking time to be rebuilt. One informant explained how his mother, who refused entry to her house of a sick neighbour at the height of the outbreak, is still shunned by that neighbour’s friends and family.

### Needs of Communities

Survivors returning from the treatment centres often struggled to reintegrate within these fractured families/communities. Whole communities felt the effects of the outbreak in a number of ways, with reduced opportunities for income generation, and consequent lack of ability to support families (including orphans), and pay for food, education and health services. Community leaders emphasised that whilst some additional resources had been provided, they were insufficient to meet the ongoing needs of the survivors and the community as a whole.

Both the community leaders and health practitioners talked a great deal about the specific ongoing needs of survivors. These were entirely consistent with the discussions within the survivors’ groups.

### Capacity to Meet Needs

Informants reflected that the authorities and national and international NGOs had provided various ways of meeting the needs of survivors, including financial support, food donations and healthcare. However, communities continue to have unmet needs, such as employment, financial stability, mental health services and social support services (such as with looking after children, orphans, and other dependents).

In some ways, the ability to provide good quality health services is stronger now, with improved knowledge, better sanitation, improved supplies and better surveillance/reporting systems. However, there are signs that some of these improvements are not being/will not be sustained. Participants explained how there had been problems with the ongoing supplies of drugs and sanitation supplies, and how the initially improved sanitation practices (such as handwashing) were not being maintained by either health workers or communities. In some ways, capacity is weaker – for instance, with rejection of health workers, affected relationships (lack of confidence) between communities and health workers, and fewer patients with the ability to pay, leading to reduced income for hospitals.

### Perspectives of Key National and International Stakeholders

#### The Needs of Survivors

Key stakeholders in general emphasised that there is a continued need for research that focuses on EVD survivors. A greater understanding of the virus itself is still required, including the risks of reinfection/transmission and the long-term health and social implications for survivors. It is clear that some survivors continue to experience stigma and discrimination leading to social isolation and loss of employment; and such exclusion can have consequences for mental health. Stakeholders responsible for large scale response and relief efforts (including Government, WHO, and UNICEF) recognised the wider, longer-term impact of the virus, but did not have the information required to understand the extent of need faced by the survivors. In addition to the potential for vulnerability to mental health problems, stakeholder participants understood that survivors may have a number of complex unmet needs, including health, psychological and social needs, and the need for assistance with community re-integration. Policy makers and providers described the importance of identifying these and other ongoing long-term needs of survivors so that they know where to focus their support now, and to be better informed for any future outbreaks. Moreover, survivors have been given a range of short-term support from the Government, as well as local and international agencies, and stakeholders felt it was important to know how that has been received and the impact that it has had.

#### Social and Economic Impact

Stakeholders also highlighted the need for research beyond the survivors, in order to more fully understand both the national and international response to the EVD epidemic in Guinea, and its wider social, economic and political impact. They described the ways in which the responses of, for example neighbouring national governments, and the international media, had sometimes profound outcomes for the people of Guinea. It was also recognised that certain aspects of the response (such as closing ports and shipping routes) could act to hinder the country’s ability to contain the outbreak (for instance, when equipment and supplies cannot easily be brought in). Discussions confirmed that it is important to make an analytical distinction between the impact of the epidemic, and the impact of the (micro, meso and macro level) responses to it, even though they are interrelated. There was clearly sometimes an element of conflict between national priorities (to contain the outbreak whilst at the same time limiting its impact on the country’s economy), and international priorities (to ensure the virus did not cross- country borders).

Informants identified the need to focus on the micro, meso and macro level responses to the epidemic and gave two reason for this. First, there was considerable variation between areas in Guinea in the disease incidence, virus transmission and the time it took to achieve containment. The reasons for this variation are not understood, yet they may hold some important lessons for improving responses to future outbreaks. Secondly, it is clear that in some communities there was resistance to the national and international response – including case management, contact tracing, sanitation practices and burial of the dead. This had important consequences for trust, community engagement and ultimately for the ability to locally contain the disease. The authorities’ initial response to the outbreak seemed to influence the ways in which the local communities reacted, affecting both disease containment and the subsequent community/family reintegration of survivors. The consideration of trust, raises the general question articulated by one informant from an academic background about whether trust relations between communities and authorities had been eroded and were at a low level prior to the epidemic, and the Ebola virus outbreak exacerbated or brought to a head the tension between the different groups or if trust relations were specifically damaged by the authorities’ response to the outbreak.

However, the key stakeholders, particularly those representing the NGOs, also emphasised that there was little evidence available about the full, wider impact of the epidemic on the country, the communities and the survivors. Key stakeholders informed us that there are a number of partial analyses of the impact of the EVD epidemic which focus on discrete areas (eg, health services, impact on economic activity) but we were not given access to these, and there appears to be no comprehensive socio-cultural, economic and policy analysis of the impact as a whole. There are also areas of impact that seem not to have been explored in the academic literature, such as: the closure of transportation routes and trade links, community cohesion, impact on religious practices and the restrictions on travel, Guinea’s capacity for research and emergency response, and the role of the global media.

#### The Need for International Comparative Research

Finally, participant stakeholders suggested that evidence from comparative research would aid the understanding of how distinctive both the response to the epidemic and its long-term impact was in Guinea. There appears to be some research collaboration between low-income countries such as Guinea, Sierra Leone and Liberia which were most affected by the outbreak, but the focus appears to be mainly on biomedical/clinical research. There is limited evidence of comparative social science research investigating, for example, variations in policy response and impact.

### Towards an Agenda for Research

The research team identified, through a thematic analysis, seven broad areas for further research that emerged from the scoping exercise. These were phrased as research questions, and discussed during a debriefing meeting with key stakeholders, who were then asked to rank them in order of priority. The priority ranking given to each key question varied considerably amongst the stakeholders, with the result that the average rankings were closely clustered. The questions, in order of average priority ranking score were:

1. What is the long-term socio-cultural, economic and health impact of the EVD epidemic on the country of Guinea?
2. What is the nature and impact of social stigma associated with EVD, and what are the factors that have contributed to the stigmatisation of survivors?
3. What can we learn from the local, national and international responses to the EVD outbreak about the nature of communication required for effective community engagement?
4. Why was the response to and effect of the Ebola virus so variable between different communities?
5. What is the impact of the EVD outbreak on non-infected community members as compared to infected survivors?
6. Are the neurological symptoms experienced by EVD survivors a consequence of direct effects of the virus, or the unmet mental health needs associated with the experience the survivors went through?
7. How did the response to and impact of the EVD outbreak vary between different countries in the region?

## Discussion

The aim of this study was to explore ideas and priorities for further health and social care research related to the EVD outbreak in Guinea from the perspective of members of the local population. A list of seven broad research questions were identified from this scoping study. However, before each of their implications are discussed, it is important to recognise the limitations of this rapid assessment.

Due to time and resource constraints, it was not possible to conduct a full research prioritisation exercise. Rather, this exercise should be seen as a pre-cursor to such a study, and results interpreted accordingly. Informants were not selected randomly but purposefully – that is, a range of people who are in an appropriate position to understand the issues, were asked to participate. The sample of key stakeholders was limited in terms of whether it fully represented the key voices in the national and local populations. It was also overwhelmingly male (all bar 2). Whilst this reflects the much smaller number of women in senior positions, it might have been possible to identify and include additional female stakeholders in a more extensive field study. The discussion groups cannot be taken to represent the beliefs of the survivors as a whole. For example, they might have been more likely to have higher levels of literacy than the general population, which is reported to have relatively low levels of literacy compared with neighbouring countries. Data collection and analysis conducted using rapid appraisal techniques may have a risk of researcher bias. This was minimised by using local ‘communicators’ (community liaison) for interviewing, as they might more easily tap into the private accounts which people would be reluctant to release to foreigners/strangers. Interviewers were given brief training by a member of the research team who is experienced in rapid appraisal methods. Analysis was conducted by an interdisciplinary team (the authors) so as to take on board a range of professional training, ethnicity, gender and theoretical perspectives.

There was evidence that some of the participants were involved in other research projects, which raises the question of whether survivors’ groups might become ‘over researched.’ However, the participants reported that whilst many had undergone repeated biological testing, this had been their first opportunity to share their thoughts, experiences and beliefs. This study began by focusing on the experiences of survivors. However, it became evident that during the course of this scoping exercise, other topic areas were identified by key stakeholders. This illustrates the flexibility and iterative nature of the methodological approach adopted but also raises the question of the extent to which the study was able to explore wider research questions when much of the focus was on survivor’s experiences. Thus, it is necessary to be cautious about the interpretation of these data particularly in terms of their transferability to other contexts.

The top priority for research for informants tended to vary according to the interests of the stakeholders. Many of the key stakeholders saw the need to assess the long-term impact of the EVD outbreak whereas, perhaps unsurprisingly, the survivors identified the question related to social stigma as being more important than did the other stakeholders. Several studies have been carried out evaluating the short-term impact of the EVD outbreak on different aspects, such as the economy and the health infrastructure.^8^ However, there has not been any comprehensive analysis of the long-term impact on Guinea as a whole, evaluating both positive and negative aspects. The field work suggested that whilst many regions of Guinea were severely affected by economic and personal loss, there are also some ways in which country capacity is now stronger, for instance for health protection and scientific research. Therefore, a systems analysis of the response and its impact could be important, utilising similar approaches and methods to those used for infectious disease preparedness or strategic planning.^30,31^ Such an analysis might probe deeper into the nature of contributory factors both for the (non)containment of the virus, and the scale of the repercussions at individual, community, country and international levels. A wide ranging and comprehensive analysis might begin by carrying out a review of the available evidence. The gaps identified in this review could then be explored through further interdisciplinary research. This analysis could provide evidence to inform policy options if there are any further epidemics of Ebola or outbreaks of similar diseases in Guinea and comparable low-income countries.

There was some consensus that survivors’ experiences need to be further investigated, although the clinical and psychiatric experience of survivors is being explored in current research carried out in the POSTEBOGUI study.^18^ More information is required about the nature and impact of social stigma, including its impact on the personal, social and economic lives of the survivors and their families. Qualitative methods might be appropriate for eliciting in-depth information about felt, enacted and courtesy stigma.^32^ This could build on and be compared with the considerable sociological research literature related to stigma and chronic illness for example, in relation to HIV/AIDS.^33^ Evidence from such research could be used to inform the development of policies aimed at enhancing the social integration of survivors, as well as national and international responses to any future epidemics.

The majority of EVD survivor studies are specifically focused on previously infected individuals. However, many non-infected members of the community have been similarly impacted, for example through financial loss, bereavement, trauma, isolation and the disruption of family and social networks. It may be beneficial to broaden the definition of ‘survivor’ to include both disease survivors as well as the non-infected survivors. By better understanding the needs of all survivors, it may be possible to identify strategies for reintegration, and for strengthening resilience within local communities.

The field work illustrated a significant number of neurological issues of unknown origin. These include symptoms such as headaches, chronic pain, fatigue, vision impairment and tremors. In addition, it seemed that survivors were suffering from a range of mental health issues that could include depression and post-traumatic stress disorder.^34^ It is not clear if these neurological issues are a direct result of viral infection or are a consequence of mental health problems associated with the outbreak. By investigating the biological persistence of the virus in the central nervous system, in conjunction with a detailed mental health assessment, it may be possible to ascertain the best ways of supporting and/or treating the survivors.

## Acknowledgements

This research prioritisation exercise was funded by a grant from the University of Kent, Kent, UK. JSR was supported by the European Research Council (FP7-PEOPLE-2012-CIG: 333955).

## Ethical issues

The study was granted ethical approval by the appropriate committee (SSPSSR) at the University of Kent, Kent, UK in November 2016. The chair of the Guinean national research ethics committee approved the conduct of the study without requiring full ethical approval in country. Informed consent (using appropriate verbal or written methods) was sought from all participants.

## Competing interests

Authors declare that they have no competing interests.

## Authors’ contributions

MC, EWG, and JSR designed and conducted the study and co-wrote the paper. MKK and AD were involved in organisation and conduct of the study, and helped with analysis of data and producing a final draft of the paper.

## Authors’ affiliations

^1^SSPSSR, University of Kent, Kent, UK. ^2^Centre for Health Services Studies, University of Kent, Kent, UK. ^3^Département Santé Publique, Université UGAN Conakry and FOSAD Health and Sustainable Development Foundation and CEFORPAG Center of Excellence for Training, Research on Malaria & Priority Diseases in Guinea, Conakry, Guinea. ^4^FOSAD Health and Sustainable Development Foundation and CEFORPAG Center of Excellence for Training, Research on Malaria & Priority Diseases in Guinea, Conakry, Guinea. ^5^School of Biosciences, University of Kent, Kent, UK.

## Key messages

**Implications for policy makers**

- The recent Ebola virus disease (EVD) epidemic in West Africa presents an important opportunity for research that will help to inform efforts to strengthen health systems, and enhance disease preparedness and control measures in the future.
- Some of the key priorities for research are to understand the long-term socio-cultural, economic and health impact of the EVD epidemic on Guinea, and to relate that to the local, national and international responses to the outbreak.
- Interdisciplinary research is required to ascertain the best ways of supporting and/or treating the survivors of EVD, and of minimising risks of future outbreaks.
- Research combining epidemiological and biological studies with a sociological analysis of community members’ beliefs and behaviours may help to develop better policies and practice for future disease containment.

**Implications for the public**

The development of intervention programmes aimed at mitigating the impact of disease epidemics need to be based on evidence derived from direct experiences of the local population. This research scoping exercise carried out in Guinea in relation to the recent Ebola epidemic identified seven research questions for further research. Each of these research questions was identified by key stakeholders and infected and non-infected members of the community and each has important implications for future disease prevention and health protection programmes. Engaging key groups in research at an early stage can help to shape the research agenda so that it is more meaningful and useful to these groups, resulting in research with greater impact.
